# Acute autonomic neuropathy with severe gastrointestinal symptoms in children: a case series

**DOI:** 10.1186/s12883-017-0943-x

**Published:** 2017-08-25

**Authors:** Ling-Yu Pang, Chang-Hong Ding, Yang-Yang Wang, Li-Ying Liu, Qiao-Jun Li, Li-Ping Zou

**Affiliations:** 1 Department of Pediatrics, Chinese PLA General Hospital, Beijing, 100853 China; 2 Department of Neurology, Beijing Children’s Hospital, The Capital Medical University, Beijing, China; 3 Department of Pediatrics, First Affiliated Hospital of the People’s Liberation Army General Hospital, Beijing, 100048 China; 4 Center of Epilepsy, Beijing Institute for Brain Disorders, Beijing, 100069 China

**Keywords:** Acute autonomic neuropathy, Children, Autonomic nervous system, Gastrointestinal dysfunction, Intravenous immunoglobulin

## Abstract

**Background:**

Acute autonomic neuropathy (AAN) is rare disorder with anecdotal report, especially for childhood onset patients. Misdiagnosis or delays in treatment can always be found in clinical practice. We conducted this study to give a description of the manifestations and treatment of AAN in children and therefore help clinicians to make the accurate diagnosis early so that the prognosis of the patients can be improved.

**Methods:**

A systematic record from 3 clinical centers was used to identify 11 subject, 3 males and 8 females, with clinical diagnosed AAN.

**Result:**

The age ranged from 2 years and 4 months to 14 years and 6 months (mean, 9 ± 3.6 years old) and the course from onset to diagnosis ranged from 7 days to 8 months. All children shared prominent initial symptoms, 7 with frequent vomiting and 4 with motor dysfunctions. The condition of 9 patients improved after treatment of IVIg and intravenous glucocorticoid.

**Conclusion:**

The clinical manifestations of AAN are diverse, generalized, and non-specific. Gastrointestinal disorders were the most common initial symptoms. Symptoms of gastrointestinal system and abnormal secretion of glands were severe and more common than other symptoms. The mechanism of AAN remains unknown. Although IVIg and intravenous glucocorticoid can be used in clinical practice, there is still no treatment recommendation and further study is needed.

## Background

Acute autonomic neuropathy (AAN), as first described in 1969, mainly involves the peripheral autonomic nervous system without other neurological manifestations [1]. The clinical features of AAN are diverse and generalized including the disturbance of pupillary constriction, gastrointestinal system, urogenital system, cardiovascular system, and gland secretion [2]. Mild sensorimotor symptoms can accompany the autonomic manifestations but not predominant [3]. . The clinical spectrum of ANN ranges from pandysautonomias involving both sympathetic and parasympathetic abnormality to merely cholinergic dysautonomias without noradrenergic dysautonomias, which overshadows the symptoms of somatic motor and sensory abnormalities.

Much as we know about the symptoms, little do we know about the pathogenesis. Some scholars believe that AAN can be considered as an uncommon variant of Guillain–Barré syndrome [3–5] with autonomic manifestations, which can result in mortality and morbidity in some patients and can also be the sole or predominant manifestation.

Most studies on AAN focus on adults instead of children [6–8]. The prominent initial symptoms are often gastrointestinal symptoms (vomiting, abdominal pain, and diarrhea). Thus, patients tend to initially be admitted to the gastroenterological department [9], which also adds the difficulty of timely diagnosis.

In this study, we described the symptoms of 11 cases in three pediatric clinical centers in China. We hope to provide more evidence for the clinical diagnosis and treatment of AAN and help improve the prognosis.

## Methods

We retrospectively investigated children with acute autonomic neuropathy diagnosed in three pediatric medical centers in China from 2003 to 2014. All clinical data were evaluated by a neurologist.

The following is the diagnostic criteria: (1) acute or sub-acute onset; (2) with clinical and electrophysiological evidence of significant autonomic failure and relative preservation of somatic motor and sensory function; (3) a self-limited course and partial recovery within 1 year to 2 years; (4) without autonomic failure because of central nervous system(CNS)disease; and (5) autonomic neuropathies of known etiology, such as those associated with amyloidosis, diabetes, malignant neoplasm, and neurotoxic drug exposure [3].

We used a series of reproducible, noninvasive, simple, and viable detection methods in the initial diagnosis and the subsequent follow-up.

1. Minor’s starch iodine test [10]: The test was administered by applying an alcohol–iodine–oil solution and starch powder. The patient received aspirin and a cup of hot water to promote sweating. The distribution of blue discoloration of the starch iodine mixture was recorded.

2. Skin scratch test [11]: The dermograph reaction indicated normal automatic nerve function if its color changes from white to red in a few minutes. Sympathetic nervous excitement was implicated if the color remained white for more than 5 min. Parasympathetic nervous excitement was implicated if the color remained red.

3. For the Lying-Standing test [12], blood pressure, electrocardiogram (ECG), and heart rate (HR) were measured as the baseline while the patient was in the supine position at resting state. All tests were repeated after standing for 10 min.

4. Oculocardiac reflex [11]: The heart rate was recorded when pressing the eyeball. The HR can decrease 10–12/min under such pressure. A sharp decrease in heart rate (more than 12/min) indicates parasympathetic hyperfunction. The absence of response to the pressure means parasympathetic inadequate function, and an increase in HR means sympathetic hyperfunction.

5. Carotid sinus reflex [11]. The heart rate in response to the pressure on the carotid sinus was recorded. The HR can decrease 6–10/min in such pressure. A sharp decrease (more than 10/min) means parasympathetic hyperfunction. The absence of response to the pressure means parasympathetic inadequate function, and an increase in HR means sympathetic hyperfunction.

The functional status of the patients was assessed in the initial and subsequent follow-up according to the modified Rankin scale [13].

## Results

### Demographics

All the 11 patients met our criteria of acute autonomic neuropathy The detailed information can be found in Table 1.

**Table 1:** Demographics and initial symptoms of the 11 patients

Case	Sex	Age	Duration*	Initial symptoms
1	F	3y2 m	38d	limb weakness
2	F	11y5m	7d	vomiting
3	M	14y6 m	3 m	lower limb weakness
4	F	8y10m	39d	limb weakness
5	F	2y4 m	25d	vomiting
6	F	8y7m	2 m	vomiting
7	F	12y	10d	vomiting
8	F	9y7m	2.5 m	vomiting
9	M	11y2 m	6 m	vomiting
10	M	7y4 m	4 m	vomiting
11	F	10y3 m	8 m	limb weakness

*represents the time from the first symptom onset to the definite diagnosis

### Clinical symptoms

The most frequent symptom is frequent vomiting, followed by motor dysfunctions. In some of them, vomiting was accompanied with lack of appetite, abdominal distention, or diarrhea. An antecedent event was reported in 9 patients before the initial symptoms of neuropathy, including gastrointestinal tract infections (*n* = 3), fever only (*n* = 3), eruption (*n* = 2), and fever with eruption (*n* = 1).

The autonomic manifestations were generalized and severe, affecting multiple systems in the body. Symptoms in the gastrointestinal system and the abnormal secretion of glands were more common than those in the other symptoms. The disturbances of the gastrointestinal system appeared in all patients and the detailed information can be found in Table 2. The weight of 10 patients decreased significantly because of malnutrition. 2 patients were tube feeding dependent.

**Table 2:** The symptoms of gastrointestinal system and glands secretion

System	N	Symptoms	n^a^ (%)
Gastrointestinal system	11 (100%)	Vomiting	8 (72.73%)
		constipation	7 (63.64%)
		Alternate of diarrhea and constipation	2 (18.19%)
		abdominal distension	2 (18.19%)
		diarrhea	1 (9.09%)
Glands secretion	11 (100%)	hyphidrosis,	10 (90.91%)
		oligosialia	7 (63.64%)
		salivate	2 (18.19%)
		hyperidrosis	1 (9.09%)
Malnutrition (marasmus)	10 (90.91%)	Severe	1 (10.00%)
		moderate	7 (70.00%)
		mild	2 (20.00%)

^a^: n/N

Severe malnutrition: weight loss >40%, moderate malnutrition: weight loss 25%–40%; mild malnutrition: weight loss 15%–25%

Symptoms also occur in other systems of the body (Table 3). In the urinary system, uroschesis was observed in 5 patients. Among them, 1 patient needed urethral catheterization because of the severely flaccid bladder. 1 patient showed delayed urination, and 1 patient showed oliguria. Symptoms in the cardiovascular system including orthostatic hypotension occurred in 4 patients. 7 patients exhibited tachycardia. Pupillary reaction to light was absent in 7 patients. Anisocoria was observed in 4 patients. Bilateral mydriasis was present in 3 patients. Abnormal shape of the pupils was observed in 1 patient. All patients showed variable somatic motor or sensory dysfunction. 8 patients experienced variable limb weaknesses. 6 patients were observed with variable loss of limb muscle strength through neurological test. Their myodynamia ranged from III to IV, as indicated by the Medical Research Council grading system (Table 3). The absence/reduction of deep tendon reflexes was observed in 7 patients. Six patients exhibited evidence of distal sensory deficit.

**Table 3:** The other symptoms of acute autonomic neuropathy

Case	Pupil	Urinary system	Orthostatic hypotension	Tachycardia	Myodynamia^aa^		Deep tendon reflexes B/P/Ac	Sensory deficits
					UL L/R	LL L/R		
1	ARL	uroschesis	−	+	IV/IV	III/III	A/A/A	−
2	anisocoria, ARL	uroschesis	Syncope	+	V^−^/V^−^	V^−^/V^−^	N/R/R	tingling
3	ARL	uroschesis	Syncope	+	*V*/V	IV/IV	N/R/R	−
4	ASP	−	Syncope	+	*V*/V	IV/IV	R/R/R	−
5	mydriasis, anisocoria, ARL	−	−	+	*V*/V	*V*/V	N/N/N	SP tenderness
6	anisocoria, ARL	delayedu- rination	Syncope	+	V^−^/V^−^	V^−^/V^−^	N/N/N	tenderness
7	−	uroschesis	−	+	III/IV	III/IV	R/A/A	−
8	mydriasis, ARL	oliguria	−	−	*V*/V	*V*/V	R/R/R	tenderness
9	ARL	uroschesis	−	−	IV/IV	IV/IV	N/N/N	numbness SP
10	mydriasis, anisocoria, ARL	−	−	−	*V*/V	*V*/V	A/A/A	tenderness
11	−	−	−	−	III/III	IV/IV	N/N/N	−

+: present; −: absence

Pupil: *ARL* = absence of reaction to light; *ASP* = abnormal shape of pupils;

Myodynamia: ^aa^ = using MRC grading system; *LL* = lower limb; L = left; *R* = right; *UL* = upper limb;

Deep tendon reflexes: *A* = absent; *Ac* = Achilles tendon; *B* = biceps tendon; *N* = normal; *R* = reduced; *P* = patellar tendon

Sensory deficits: SP = spontaneous pain

### Autonomic function tests

Minor’s starch iodine test was performed in 7 patients. 6 of them showed significant areas of anhidrosis, whereas 1 showed hidrosis in the feet and buttocks. The skin scratch test result was abnormal in 8 patients. Oculocardiac and carotid sinus reflexes were performed in 5 patients. However, the HR showed no change in 4 and increment in 1. The result of Lying–Standing test was positive in 4 patients.

### Gastrointestinal studies

Gastrointestinal barium meal examination was performed in 2 patients (patients 1 and 2). Patient 1 showed enteroplegia and secondary megacolon. Patient 2 showed incomplete distal esophageal obstruction and cardiospasm. Gastroscopy examination was performed in patient 9 and showed esophageal and gastric ulcers. Chest computerized tomography (CT) scan examination was performed in 2 patients and showed megaesophagus and cardiospasm. 8 patients were found to have low intestinal obstruction by abdominal and pelvic CT scan examination.

### Electrophysiological studies

The electrophysiological examination results of 10 patients were summarized in Table 4. The motor nerve conduction was abnormal in 2 patients. The sensory nerve conduction was abnormal in 3 patients. 8 patients showed nerve damages in needle electromyography examination. Fibrillation potentials and positive waves were present in 3 patients in mild muscle contraction. 2 patients showed monophasic pattern in strong muscle contraction.

**Table 4:** The results of nerve conduction and needle EMG

MCV (n, %)							SCV (n,%)							Needle EMG (n,%)				
Nerve	n	CV			AMP		Nerve	n	CV			AMP		Muscle	n	Prolongation of MUAP duration	FP, PSP	Abnormal RP
		N	R		N	R			N	R		N	R					monophase
CPN	10	10 (100%)	0		10 (100%)	0	SN	10	8 (80%)	2 (20%)		9 (90%)	1 (20%)	BB	10	2 (20%)	3 (30%)	2 (20%)
TN	10	9 (90%)	1 (10%)		10 (100%)	0	MN	10	9 (90%)	1 (10%)		8 (80%)	2 (20%)	ATM	8	1 (12.5%)	3 (37.5)	2 (25%)
MN	10	9 (90%)	1 (10%)		9 (90%)	1 (10%)	UN	10	9 (90%)	1 (10%)		8 (80%)	2 (20%)	QFM	9	2 (22.22%)	3 (33.33%)	2 (22.22%)
UN	10	9 (90%)	1 (10%)		9 (90%)	1 (10%)								EDCM	9	3 (33.33%)	1 (11.11%)	1 (11.11%)

*AMP* Amplitude, *ATM* anterior tibial muscle, *BB* biceps brachii, *CPN* common peroneal nerve, *CV* conduction velocity, *EDCM* extensor digitorum communis muscle, *EMG* electromyogram, *FP* fibrillation potential, *MCV* motor conduction velocity, *MN* median nerve, *MUAP* motor unit action potential, *N* normal, *PSP* positive shape potential, *QFM* quadriceps femoris muscle, *R* reduced, *RP* recruitment pattern, *SCV* sensory conduction velocity, *SN* sural nerve, *TN* tibial nerve, *UN* ulnar nerve

The ECG results showed sinus tachycardia in 10 cases, P-R intervals of mild extension in 2 cases, and high-peaked P wave in 2 cases.

### Laboratory investigations

The cerebrospinal fluids (CSF) were obtained in 10 of 11 patients. The CSF protein level was elevated in 3 patients (patients 2, 3, and 4). Patient 9 showed dissociation of protein from cell in CSF. 2 patients displayed oligoclonal band in CSF. In one patient, only the value of CSF-IgG elevated, and in another patient, the value of CSF-IgG, IgA, and IgM elevated simultaneously. Infection markers were detected in 2 patients. 1 patient showed C-reactive protein increasing and 1 patient showed elevating erythrocyte sedimentation rate. 1 case was positive for herpes simplex virus IgM antibody. Another was simultaneously positive for Epstein Barr Virus and herpes simplex virus antibody. No other serological or etiological abnormality was noted in laboratory studies.

### Treatment and follow-up

The treatments of each patient were listed in Table 5. IVIg combined with intravenous glucocorticoid (methylprednisolone or dexamethasone) was administered to 8 patients. 4 of these patients were additionally administered with gabapentin. Patients 1 and 3 were treated with IVIg alone, whereas patient 6 received other symptomatic treatment. Patients administered with intravenous glucocorticoid were subsequently given glucocorticoid orally. In addition, supportive treatment was administered to all the patients with gastrointestinal symptoms. These treatments included omeprazole to suppress gastric acid secretion, vitamin B6 to ease vomiting, Marzulene-S granules (l-glutamine and azulene sodium sulfonate) to protect the gastric mucosa, and domperidone or mosapride to promote gastrointestinal motility. Four patients were severely ill, and their functional status was 5 at peak phase. 2 of these patients died within 1 year since the onset of AAN. Cyclophosphamide was administered in patient 11 as symptoms continued to exacerbate during treatment. A slight alleviation of autonomic symptoms was noted in patient 9 after 1 year of follow-up. The functional status of 8 patients was 4 at peak phase. Gradual recovery, especially autonomic symptoms, was observed in these patients after several months of follow-up.

**Table 5:** The treatment and follow-up

Case	Treatment	Modified Rankin Scale		
		peak	discharged	1 year
1	IVIg	4	3	ND
2	IVIg, IVGC	5	5	Dead
3	IVIg	4	4	3
4	IVIg, IVGC	4	3	1
5	IVIg, IVGC	4	3	2
6	ST	4	3	ND
7	IVIg, IVGC	5	4	ND
8	IVIg,IVGC, gabapentn	4	3	1
9	IVIg,IVGC, gabapentn	5	5	4
10	IVIg,IVGC, gabapentn	4	3	1
11	IVIg,IVGC Cyclophosphamide	5	5	Dead

*IVIg* intravenous immunoglobulin, *IVGC* intravenous glucocorticoid, *ND* not determin

The autonomic abnormalities of different systems recovered differently. Gastrointestinal symptoms were difficult to alleviate, and with high recurrence rate. Abnormal secretion of glands and sensory deficits recovered significantly, although they tended to persist for a long time. Urinary system dysfunction stopped progressing after the initial treatment and recovered completely in the end. 2 patients showed complete recovery from orthostatic hypotension at discharge. The other 2 patients exhibited mild improvement.

## Discussion

Gastrointestinal dysfunction was the most common initial symptom. Most patients suffered from gastrointestinal dysfunction especially unexplained vomiting at the onset of this disease. These symptoms showed little improvement even after symptomatic treatment in gastrointestinal department. Some even exacerbated. Most patients presented significant weight loss and suffered from severe malnutrition, even cachexia. Besides, although the initial symptoms of some patients were limb weaknesses, gastrointestinal symptoms appeared and gradually became prominent subsequently. Early diagnosis was difficult through the course of the disease because of the lack of featured symptoms. The duration from onset to diagnosis was more than one month in most patients. The longest duration was eight months. We hope that pediatric gastroenterologist may notice some subtle difference between these symptoms and their classic cases. Therefore, patients can seek for help from specialists earlier.

The main clinical manifestation of AAN is autonomic dysfunction with two main aspects—cholinergic and noradrenergic abnormalities. In our patients, the former is featured with parasympathetic disorder, involving gastrointestinal dysfunction, loss of pupillary constriction, atony of the bladder, impaired salivation, and sweating. The unchanged HR in oculocardiac reflex test and carotid sinus reflex test can also indicate its existence. The latter is featured with orthostatic hypotension and positive Lying–Standing test, and it has been found in 4 patients who also presented cholinergic abnormities. The other 7 patients only presented cholinergic abnormalities, which conformed to the typical manifestations of AAN with only cholinergic autonomic dysfunction [14]. Some literatures have pointed that acute cholinergic dysfunction particularly affected younger people [15].

According to the literature, some patients exhibited somatic motor or sensory dysfunction [16]. A few patients complained of limb weakness, distal muscular atrophy, and distal limb sensory disturbance [17]. Distal sensory deficits and variable limb weaknesses were also observed in our study. Nerve conduction was normal in most patients. Needle electromyography showed nerve damages in 8 patents. Some studies revealed that the lesion is peripheral [1]. Thus, we suspected that the reason of motor dysfunction may be dystrophia of muscular tissue caused by denervation. 10 patients showed malnutrition even cachexia and this may be the reason of increasing motor dysfunction.

The treatment of AAN to date is mainly immune regulation involving IVIg and plasma exchange. Meanwhile, supportive treatment remains the cornerstone of management. IVIg treatment was very effective not only for sensory and motor symptoms but also for autonomic symptoms. 10 patients were treated with IVIg in this study. 8 of these patients were given additional intravenous injection of glucocorticoid. The symptoms still progressed in 4 patients even under immunomodulatory treatment. Some patients needed repetitive therapy. 2 of these patients died of severe malnutrition. Progression was ceased in other patients after the treatments. The sensory and motor symptoms recovered significantly, whereas partial autonomic symptoms tended to persist for a long time without interfering daily life.

The limitation of our study was that our sample came from the hospitalized patients, and the characteristics of this sample might be different from the overall characteristics of the affected population. Thus, more systemic studies are needed to help clinicians understand this disease.

## Conclusions

The clinical manifestations of AAN are diverse, generalized, and non-specific. Gastrointestinal disorders were the most common initial symptoms. Symptoms of digestive system and abnormal secretion of glands were severe and more common than other symptoms. Most patients suffered from moderate or severe malnutrition. Cholinergic autonomic nervous functions were often disturbed in affected children. Most patients made favorable progress after treatment with IVIg combined with intravenous glucocorticoid. The mechanism of AAN remains unknown. The treatment recommendation is also not available. Further exploration is thus needed.

## Abbreviations

AAN: Acute autonomic neuropathy
CNS: Central nervous system
CSF: The cerebrospinal fluids
ECG: Electrocardiogram
HR: Heart rate
IVIG: Intravenous immunoglobulin

## References

[1] Suarez GA, Fealey RD, Camilleri M, Low PA (1994). Idiopathic autonomic neuropathy: clinical, neurophysiologic, and follow-up studies on 27 patients. Neurology.

[2] McDougall AJ, McLeod JG (1996). Autonomic neuropathy, I. Clinical features, investigation, pathophysiology, and treatment. J Neurol Sci.

[3] Freeman R (2005). Autonomic peripheral neuropathy. Lancet.

[4] Feldman EL, Bromberg MB, Blaivas M, Junck L (1991). Acute pandysautonomic neuropathy. Neurology.

[5] Ishitobi M, Haginoya K, Kitamura T, Munakata M, Yokoyama H, Iinuma K (2004). Acute dysautonomia: complete recovery after two courses of IVIg. Brain and Development.

[6] Besnard M, Faure C, Fromont-Hankard G (2000). Intestinal pseudo-obstruction and acute pandysautonomia associated with Epstein-Barr virus infection. Am J Gastroenterol.

[7] Clark MB, Davis T (2013). A pediatric case of severe pandysautonomia responsive to plasmapheresis. J Child Neurol.

[8] Hanai S, Komaki H, Sakuma H (2011). Acute autonomic sensory and motor neuropathy associated with parvovirus B19 infection. Brain and Development.

[9] Koike H, Atsuta N, Adachi H (2010). Clinicopathological features of acute autonomic and sensory neuropathy. Brain.

[10] Choi HG, Kwon SY, Won JY (2013). Comparisons of three indicators for Frey's syndrome: subjective symptoms, Minor's starch iodine test, and infrared thermography. Clin Exp Otorhinolaryngol.

[11] Jiang Wu JJ (2013). LiYing cui Neurology.

[12] Futang Zhu YH, Jiang Z (2002). Textbook of pediatrics.

[13] van Swieten JC, Koudstaal PJ, Visser MC, Schouten HJ, van Gijn J (1988). Interobserver agreement for the assessment of handicap in stroke patients. Stroke.

[14] Takayama H, Kazahaya Y, Kashihara N (1987). A case of postganglionic cholinergic dysautonomia. J Neurol Neurosurg Psychiatry.

[15] Hart RG, Kanter MC (1990). Acute autonomic neuropathy. Two cases and a clinical review. Arch Intern Med.

[16] Low PADP, Lambert EH (1983). Acute panautonomic neuropathy. Ann Neurol.

[17] McLeod JG (1992). Invited review: autonomic dysfunction in peripheral nerve disease. Muscle Nerve.

